# Comparison of Postoperative Events between Spinal Anesthesia and General Anesthesia in Laparoscopic Cholecystectomy: A Systemic Review and Meta-Analysis of Randomized Controlled Trials

**DOI:** 10.1155/2016/9480539

**Published:** 2016-07-25

**Authors:** Xian-Xue Wang, Quan Zhou, Dao-Bo Pan, Hui-Wei Deng, Ai-Guo Zhou, Hua-Jing Guo, Fu-Rong Huang

**Affiliations:** ^1^Department of Anesthesiology, The First People's Hospital of Changde City, Changde, Hunan 415003, China; ^2^Science & Education Division, The First People's Hospital of Changde City, Changde, Hunan 415003, China

## Abstract

*Background*. Laparoscopic cholecystectomy is usually carried out under general anesthesia. There were a few studies which have found spinal anesthesia as a safe alternative. We aimed to evaluate the postoperative events between spinal anesthesia and general anesthesia in patients undergoing laparoscopic cholecystectomy.* Methods*. We searched PubMed, Embase, and Cochrane Library (from inception to January 2016) for eligible studies. The primary outcome was the visual analogue scale score. Secondary outcomes included postoperative nausea and vomiting and urine retention 24 hours postoperatively. We calculated pooled risk ratios and 95% confidence interval using random- or fixed-effects models.* Results*. Eight trials involving 723 patients were listed. Meta-analysis showed that patients in spinal anesthesia groups have lower visual analogue scale score 24 hours postoperatively. There were significant decreases in the occurrence of postoperative nausea and vomiting in spinal anesthesia group when compared with general anesthesia group (odds ratios: 0.38, 95% confidence interval: 0.19–0.76; *P* = 0.006) with heterogeneity accepted (*I* ^2^ = 13%; *P* = 0.33), while urine retention rate was increased in patients with spinal anesthesia (odds ratios: 4.95, 95% confidence interval: 1.24–19.71; *P* = 0.02) without any heterogeneity (*I* ^2^ = 0%; *P* = 0.98).* Conclusions*. Spinal anesthesia may be associated with less postoperative pain and postoperative nausea and vomiting compared with general anesthesia.

## 1. Introduction

Laparoscopic cholecystectomy (LC) was the gold standard for the surgical treatment of symptomatic cholelithiasis [1]. It may be awarded to minimally invasive nature of the procedure and associated with less postoperative pain, reduced hospital stay, and earlier return to daily activities [2, 3].

LC is performed under general anesthesia and may be involved in postoperative pain and nausea and vomiting (PONV). Spinal anesthesia was a less invasive anesthetic technique that has lower morbidity and mortality rates, compared with general anesthesia [4]. Patients who received spinal anesthesia undergoing laparoscopy interventions were usually awake, felt less pain, and tended to ambulate earlier with no intubation and/or extubation [5, 6].

Combining a minimally invasive surgical procedure with a less invasive anesthetic technique, spinal anesthesia seems to further enhance the advantage of LC. Despite the mentioned advantage, the use of spinal anesthesia for LC has still not gained popularity. Recently, some studies have highlighted the feasibility and safety of performing LC under spinal anesthesia. The purpose of this study was to compare the postoperative effects between spinal anesthesia and general anesthesia in patients undergoing LC.

## 2. Methods

This systematic review was conducted according to the guidelines of the preferred reporting items for systematic reviews and meta-analyses (PRISMA) [7]. We prospectively registered our system review at PROSPERO (registration number: CRD42015017169). Our study is a meta-analysis, so ethics approval and consent are not required.

### 2.1. Data Sources and Search Strategy

PubMed, Embase, and Cochrane Library databases were searched from inception to January 2016 for relevant studies investigating the effect between spinal anesthesia and general anesthesia. The following search terms were used: general anesthesia, “anesthesia, general”, spinal anesthesia, “anesthesia, spinal”, “cholecystectomy, laparoscopy”, laparoscopy cholecystectomy, “cholecystectomy, celioscopic”, “cholecystectomies, celioscopy”, and celioscopy cholecystectomy. A hand search in reference sections of included trials, published meta-analyses, and relevant review articles was conducted to identify additional articles. If duplicated data were presented in several studies, only the most recent, largest, or most complete study was included.

### 2.2. Study Selection

Original studies included were based on PICOS (patient, intervention, comparison, outcome, and study design) as follows: (a) P, American Society of Anesthesiology (ASA) I/II grade adult patients undergoing laparoscopy cholecystectomy; (b) I and C, spinal anesthesia and general anesthesia, respectively; (c) O, pain score, the incidence of PONV, and urine retention; (d) S, only randomized controlled trials (RCTs) included. Only English was set.

### 2.3. Data Extraction

Characteristics of patients (number of patients, American Society of Anesthesiologist (ASA) rating, age, gender, and type of surgery and anesthesia) and trials design (intervention, follow-up time, and reported outcomes) were also recorded. If the data mentioned above were unavailable in the article, the corresponding authors were contacted for missing information. All data were independently extracted using a standard data collection form by 2 reviewers (Xian-Xue Wang and Quan Zhou), and then the collected data were checked and entered into Review Manager analyses software (RevMan) version 5.3. All discrepancies were checked and a consensus was reached by discussion with a third author (Dao-Bo Pan) involved. A record of reasons for excluding studies was kept.

### 2.4. Assessment of Study Quality

A critical evaluation of the included studies quality was performed by 2 reviewers (Xian-Xue Wang and Quan Zhou) by using a 5-point Jadad scale [8]. The main categories consisted of the following 5 items: “was the study described as randomized? (1),” “was the method used to generate the sequence of randomization described and appropriate (random numbers, computer-generated, etc.)? (1),” “was the study described as double-blind? (1),” “was the method of double-blinding described and appropriate (identical placebo, active placebo, dummy, etc.)? (1),” and “was there a description of withdrawals and dropouts? (1).” A score of 4 to 5 was considered a high methodological quality.

### 2.5. Assessment of Risk of Bias

Two reviewers (Xian-Xue Wang and Quan Zhou) independently evaluated the risk of bias according to the recommendations from the Cochrane Collaboration [9]. The main categories consisted of random sequence generation, allocation concealment, blinding of participants and personnel, blinding of outcome assessment, incomplete outcome data, selective reporting, and other biases. Each domain was assessed as “high risk,” “low risk,” or “unclear risk.” Namely, the judgment was “low risk” for the item with sufficient and correct information. And the judgment was “high risk” for the item reported incorrectly. If the information of the item was insufficient or unsanctioned, the judgment was “unclear risk.” An “unclear risk” judgment should also be made if the item was reported, but the risk of bias is unknown. The disagreement was solved by a senior reviewer (Dao-Bo Pan).

### 2.6. Statistical Analysis

Odds ratio (OR) or weighted mean difference (WMD) with 95% confidence interval (CI) was used as a common measure of the effect between spinal anesthesia and general anesthesia. *I* ^2^ value was used to estimate statistical heterogeneity. When *I* ^2^ < 50%, heterogeneity could be accepted and the fixed-effects model was adopted. Otherwise, the randomized-effects model was adopted and sensitivity analysis used. Whenever heterogeneity was present, several sensitivity analyses were carried out to identify potential sources. We also investigated the influence of a single study on the overall pooled estimate by omitting one study in each turn. Owing to the limited number (below 10) of studies included in each analysis, publication bias was not assessed. A *P* value < 0.05 was considered statistically significant. Risk-of-bias assessment was conducted by using Review Manager, version 5.3 (the Cochrane Collaboration, Software Update, Oxford, UK). Power analyses of individual studies and meta-analysis were all conducted by the software, version 4.1.0.

## 3. Results

### 3.1. Identification of Eligible Studies

**Figure 1:**
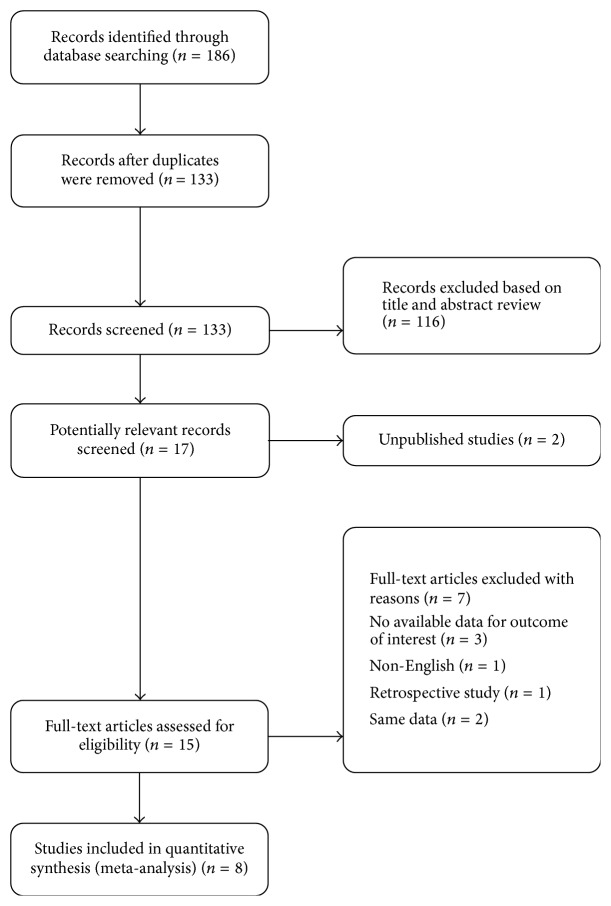
Flow diagram of search strategy and study selection.

A total of 186 potentially relevant abstracts were detected. After duplicates were deleted, one hundred and thirty-three unique abstracts remained. After looking at the abstracts, seventeen publications seemed to meet the inclusion criteria. For the remaining 17 articles, nine of them were excluded for the following reasons: unpublished studies, no available data on the outcome of interest in [15–17], non-English [14], retrospective study [18], and same data [19, 20]. Finally, the remaining 8 studies [1, 4, 10–14, 21] with existing data met our selection criteria and were included in the systematic review. The flow diagram of search strategy and study selection was shown in Figure 1.

### 3.2. Study Characteristics

The characteristics of all included studies were submitted in Table 1. All were adult patients undergoing LC. High Jadad score of the studies included was 7 (range from 4 to 5). Publication bias was not studied.

These studies were expected to be released between 2008 and 2014. Sample size of included studies ranged from 20 to 224. All were randomized controlled trials and primary end points were VAS, PONV, and urine retention. One study compared epidural anesthesia with general anesthesia in patients undergoing LC [21]. Intra-abdominal carbon dioxide pressure was lower in all studies: four studies with carbon dioxide at maximum intra-abdominal pressure of 10 mmHg and one study set below 8 mmHg. Carbon dioxide pneumoperitoneum pressure was from 12 to 15 mmHg in 3 studies. No significant side effects were observed between spinal anesthesia and general anesthesia groups (Table 1).

### 3.3. VAS Score 2 Hours Postoperatively

**Figure 2:**
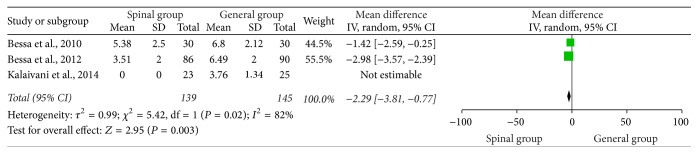
VAS 2 hours postoperatively.

Three studies have examined the VAS 2 hours postoperatively [1, 4, 12]. The aggregated results of these four studies suggest that the VAS score in the spinal group was associated with a significant reduction compared with the general group (WMD = −2.29, 95% CI: −3.81 to −0.77, *P* = 0.003) (Figure 2). Heterogeneity was noted among the studies (*I* ^2^ = 82%; *P* = 0.02); randomized-effects model was selected.

### 3.4. VAS Score 4 Hours Postoperatively

**Figure 3:**
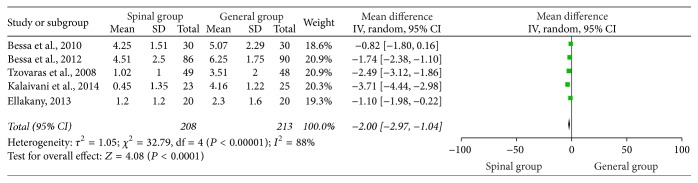
VAS 4 hours postoperatively.

VAS 4 hours postoperatively was investigated in 5 trials [1, 4, 11–13]. Compared with general group, VAS score in the spinal group was statistically significantly reduced (WMD = −2.00, 95% CI: −2.97 to −1.04, *P* < 0.0001) (Figure 3). Heterogeneity was observed among the studies (*I* ^2^ = 88%; *P* < 0.00001). Subsequently, we performed sensitivity analyses to consider potential sources of heterogeneity. Exclusion of any single study did not resolve the heterogeneity; thus, randomized-effects model was selected.

### 3.5. VAS Score 8 Hours Postoperatively

**Figure 4:**
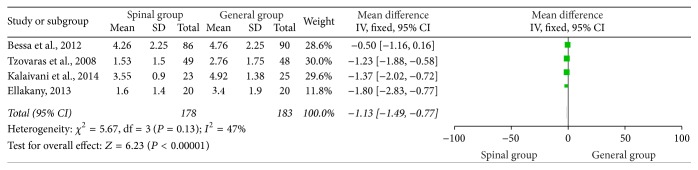
VAS 8 hours postoperatively.

As shown in Figure 4, four studies [1, 11–13] were incorporated into the meta-analysis. VAS score in the spinal group 8 hours postoperatively was significantly reduced (WMD = −1.13, 95% CI: −1.49 to −0.77, *P* < 0.00001) (Figure 4). Heterogeneity was accepted among the studies (*I* ^2^ = 47%; *P* = 0.13); fixed-effects model was selected.

### 3.6. VAS Score 24 Hours Postoperatively

**Figure 5:**
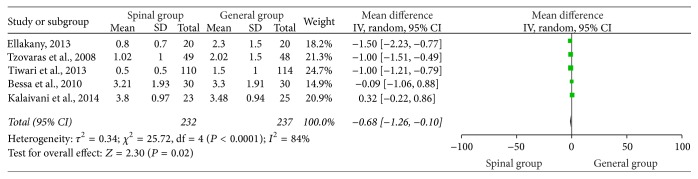
VAS 24 hours postoperatively.

**Figure 6:**
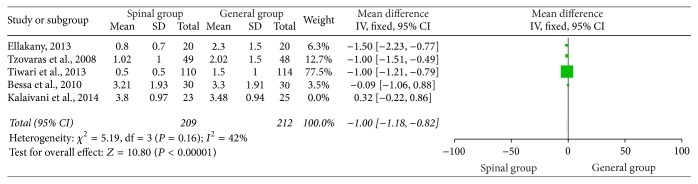
VAS 24 hours postoperatively after sensitivity analyses were performed.

Figure 5 outlines the VAS score 24 hours postoperatively. The results of these studies [4, 10–13] suggested that the VAS score was significantly reduced in the spinal group when compared with the general group (WMD = −0.68, 95% CI: −1.26 to −0.10, *P* < 0.02) (Figure 5). Subsequently, we performed sensitivity analyses to consider potential sources of heterogeneity. Exclusion of the trial conducted by Tiwari et al. [10] resolved the heterogeneity but did not change the results (WMD = −1.00, 95% CI: −1.18 to −0.82, *P* < 0.00001; *P* for heterogeneity = 0.16; *I* ^2^ = 47%) (Figure 6). Further exclusion of any single study did not materially change the overall combined RR.

### 3.7. Postoperative Nausea and Vomiting within 24 Hours

**Figure 7:**
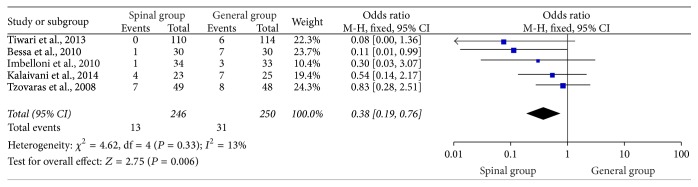
Postoperative nausea and vomiting within 24 hours.

In total, 7 studies [1, 4, 10–12, 14, 21] provided evidence on the incidence of postoperative nausea and vomiting (PONV) between spinal group and general group. One study [1] compared the incident rates of PONV at 8 hours and found the incidence rate significantly decreased in the spinal group (6.9% in spinal group versus 22.2% in general group; *P* = 0.004), while another study [21] found that one patient in spinal group versus three patients in general group experienced nausea, which is not statistically significant between the two groups. The remaining 5 studies [4, 10, 11, 13, 22] reported PONV during a period of 24 hours and could perform meta-analysis.  Figure 7 outlines the PONV within 24 hours. The results of these studies suggested that the overall PONV rate significantly reduced in the spinal group (OR: 0.38, 95% CI: 0.19–0.76; *P* = 0.006) with heterogeneity accepted (*I* ^2^ = 13%; *P* = 0.33).

### 3.8. Postoperative Urine Retention within 24 Hours

**Figure 8:**
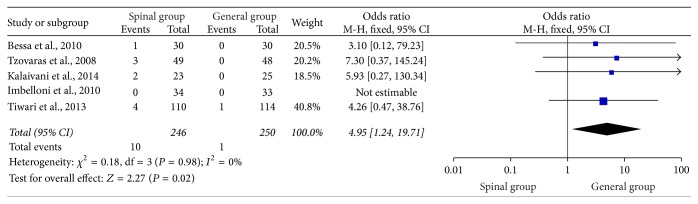
Urine retention 24 hours postoperatively.

Seven studies [1, 4, 10–12, 14, 21] provide evidence on the incidence of postoperative urine retention between spinal group and general group. Two studies [1, 21] compared the incident rates of urine retention (at 2 and 8 hours, resp.) and found no statistically significant difference between spinal group and general group (*P* > 0.05). The remaining 5 studies [4, 10, 11, 13, 22] reported urine retention during a period of 24 hours and could perform meta-analysis. As shown in Figure 8, the results of these studies suggested that the overall urine retention rate significantly increased in the spinal group (OR: 4.95, 95% CI: 1.24–19.71; *P* = 0.02) without any heterogeneity (*I* ^2^ = 0%; *P* = 0.98).

### 3.9. Quality Assessment

**Figure 9:**
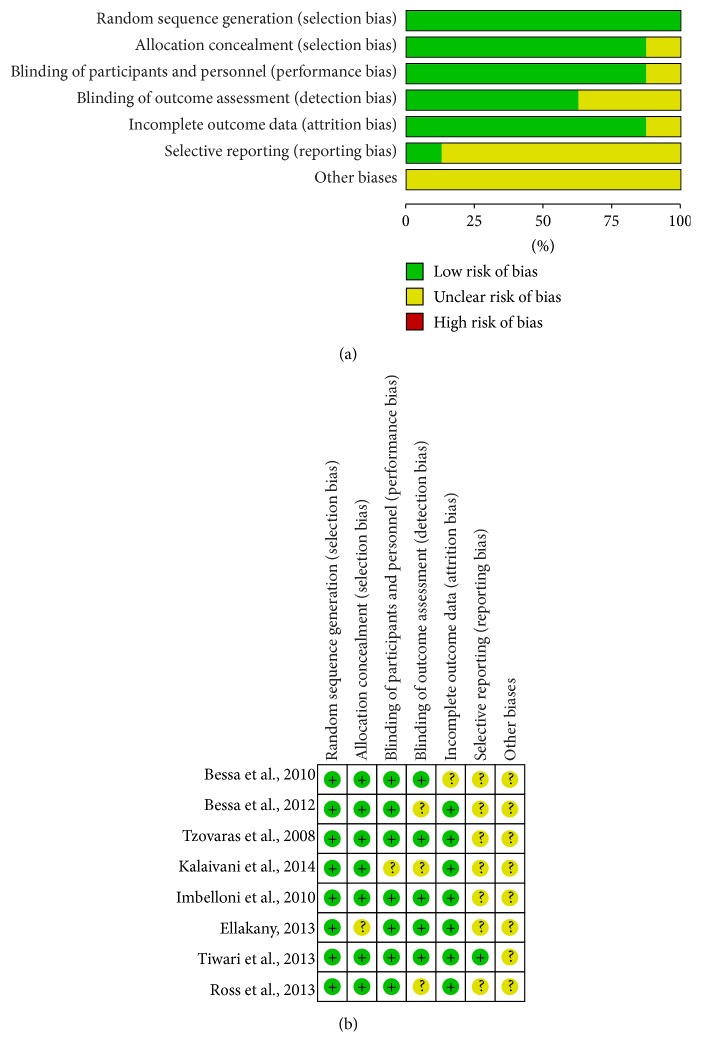
(a) Risk of bias graph. (b) Risk of bias summary.

This systematic review included 8 RCTs: the baseline characteristics of patients were reported in all trials, and four trials mentioned the method of randomness (Figure 9).

### 3.10. Power Analysis

Although the statistical results were indicated in some studies, a portion of the primary data was unavailable. The available data were reassessed by a power analysis with an alpha level of 0.05 (Table 2). The power of an individual study ranged from 5% to 100%. The power of the meta-analysis with respect to VAS score (2 hours, 4 hours, 8 hours, and 24 hours), PONV, and urine retention, respectively, is shown in Table 2. Eleven of 23 outcomes were less than 50%, while twelve outcomes were larger than 80%.

## 4. Discussion

In the present meta-analysis, we have reviewed and considered the literature regarding the efficacy between spinal anesthesia and general anesthesia in decreasing postoperative VAS score, PONV, and urine retention in adult patients undergoing LC. The pooled results from meta-analysis of seven RCTs using both random-effects and fixed-effects model suggest that spinal anesthesia shows beneficial efficacy in preventing postoperative pain and PONV in adult patients undergoing LC, while more patients exhibit urine retention in the spinal group comparing with the general group. Also, substantial heterogeneity across the studies was observed.

LC, considered a minimally invasive surgery, is usually done under general anesthesia. Our meta-analysis showed that there are certain indications for spinal anesthesia in patients undergoing elective LC. Regional anesthesia reduced the surgical stress response. In spinal anesthesia, there was no airway instrumentation and there was a low incidence of deep vein thrombosis [22]. Our study suggested that patients in the spinal group experienced significantly less pain postoperatively. There were two reasons for the reduced pain in the spinal anesthesia group. One was the persistent neuraxial blockade by spinal anesthesia, and it may be the lower VAS score in the spinal group. The other factor may be the point of low-pressure pneumoperitoneum. A recent meta-analysis suggested that the use of low-pressure pneumoperitoneum seems to be effective in reducing pain after LC [23]. PONV, a complex multifactorial problem, is more frequent after general anesthesia compared with spinal anesthesia. Sinha et al. [18] reported PONV rates of 2% in spinal anesthesia patients undergoing LC, while 29% of patients in the general anesthesia group experienced PONV. For reduced PONV, a nasogastric tube was inserted usually in general patients undergoing LC to deflate the stomach and allow for better exposure of the operative field, but this is not required in patients who have received spinal anesthesia [11, 22].

In our meta-analysis, the power ranged from 5% to 100% and 12 of 23 outcomes were larger than 80%. Power of 8 outcomes was lower than 50% and the lowest power came from the 24-hour postoperative VAS score in Bessa et al.'s study [4]. All the results tell us that there is no sufficient evidence on the effects and high level studies are still needed [24–26].

To our knowledge, this is the first meta-analysis to compare the application of spinal anesthesia with general anesthesia in patients who undergo elective LC. Spinal anesthesia is feasible and safe for patients undergoing LC in low-pressure pneumoperitoneum and may be more effective than the general anesthesia. From these data, it appears that postoperative VAS score of patients in spinal group was significantly lower when compared with general group and postoperative pain was lighter. And the occurrence rate of PONV in spinal group was lower. At the same time, there was no airway instrumentation and surgical stress response reduced in spinal group. Moreover, the cost of spinal anesthesia was lower. So spinal anesthesia may be a promising method of anesthesia for laparoscopy procedure, while postoperative urine retention rate was higher in the spinal anesthesia group when compared with general anesthesia group. It seems to be a significant factor to evaluate the method of spinal anesthesia in patients undergoing LC.

In our meta-analysis, all the studies matched well (e.g., sex, age, ASA grade, administration time, and way of surgery), while several limitations should be taken into account. First, the articles included in our meta-analysis mostly focus on postoperative indexes. In order to comprehensively evaluate the advantage of spinal anesthesia, some intraoperative indexes should be compared between two groups, such as pull reaction of patients and hemodynamic indexes during operation and during surgery. Only three studies [12, 14, 21] pay attention to the time of surgery; all of them found that there was no statistical significance between the two groups. Second, the intra-abdominal pressures among the articles of our study are different and have no unified standards, which may lead to bias of results. The duration of postoperative pain also should be concerned in the two groups and prospective studies to see the duration of analgesia of spinal anesthesia are needed. Third, VAS was based on subjective feeling and default objective standard, resulting in possible overestimation or underestimation of the true effect of spinal anesthesia compared with general anesthesia. Fourth, our team mainly focused on studies published in the English language and bias might be existent. The sample sizes of individual trials included were small or moderate, which may be the reasons for lower power in some study outcomes. The study number was below 10, which is not statistically significant to assess publication bias.

Spinal anesthesia may be associated with less postoperative pain and PONV compared with general anesthesia. Considering the limitations above, our finding should be elucidated carefully and large-scale studies were needed in order to confirm it.

## Figures and Tables

**Table 1:** Characteristics of trials included in systematic review.

Study	Number of patients (spinal anesthesia/general anesthesia)	Study design	Intra-abdominal CO_2_ pressure	Patient characteristics	Primary end point	Jadad score
Tiwari et al., 2013 [10]	110/114	RCT	8–10 mmHg	Adult patients undergoing laparoscopic cholecystectomy	Pain score (VAS), nausea/vomiting, urinary retention, hypotension, headache, back pain, sore throat	5
Bessa et al., 2012 [1]	86/90	RCT	10 mmHg	Adult patients undergoing laparoscopic cholecystectomy	Pain score (VAS), PONV, urine retention, postoperative spinal headache, wound sepsis	4
Tzovaras et al., 2008 [11]	49/48	RCT	≤10 mmHg	Adult patients undergoing laparoscopic cholecystectomy	Pain score (VAS), PONV, urinary retention, dizziness	5
Kalaivani et al., 2014 [12]	23/25	RCT	12 mmHg	Adult patients undergoing laparoscopic cholecystectomy	Pain score (VAS), PONV, urinary retention, postoperative spinal headache, wound sepsis	3
Bessa et al., 2010 [4]	30/30	RCT	≤15 mmHg	Adult patients undergoing laparoscopic cholecystectomy	Pain score (VAS), PONV, urine retention, postoperative spinal headache, wound sepsis	4
Ellakany, 2013 [13]	20/20	RCT	≤10 mmHg	Adult patients undergoing laparoscopic cholecystectomy	Pain score (VAS), patient satisfaction, surgeon satisfaction	4
Imbelloni et al., 2010 [14]	34/33	RCT	≤8 mmHg	Adult patients undergoing laparoscopic cholecystectomy	Pain score (VAS), PONV, urine retention, shoulder pain, pruritus, duration of the sensorial blockade, duration of the motor blockade	5
Ross et al. 2013 [21]	10/10	RCT	12–15 mmHg	Adult patients undergoing laparoscopic cholecystectomy	Pain score (VAS), shoulder pain, nausea, urinary retention, severe abdominal pain, dizziness	5

PONV: postoperative nausea and vomiting.

**Table 2:** Power analysis of the studies.

Study	VAS (2 h)	VAS (4 h)	VAS (8 h)	VAS (24 h)	PONV (24 h)	Urine retention (24 h)
Bessa et al., 2010 [4]	70%	37%	NA	5%	64%	8%
Bessa et al., 2012 [1]	100%	100%	32%	NA	NA	NA
Kalaivani et al., 2014 [12]	100%	100%	99%	33%	15%	21%
Tzovaras et al., 2008 [11]	NA	100%	95%	97%	8%	27%
Ellakany, 2013 [13]	NA	67%	91%	98%	NA	NA
Tiwari et al., 2013 [10]	NA	NA	NA	100%	82%	19%
Imbelloni et al., 2010 [14]	NA	NA	NA	NA	18%	NA

NA: not available.
